# Metformin as adjunctive therapy for dengue in overweight and obese patients: a protocol for an open-label clinical trial (MeDO)

**DOI:** 10.12688/wellcomeopenres.16053.2

**Published:** 2021-02-08

**Authors:** Nguyet Minh Nguyen, Ho Quang Chanh, Dong Thi Hoai Tam, Nguyen Lam Vuong, Nguyen Thi Xuan Chau, Nguyen Van Vinh Chau, Nguyen Thanh Phong, Huynh Trung Trieu, Tai Luong Thi Hue, Tam Cao Thi, Trung Dinh The, Huynh Thi Le Duyen, Ninh Thi Thanh Van, Quyen Nguyen Than Ha, Laura Rivino, Peter Gallagher, Nick K. Jones, Ronald B. Geskus, Evelyne Kestelyn, Sophie Yacoub

**Affiliations:** 1 Oxford University Clinical Research Unit, Ho Chi Minh City, 700000, Vietnam; 2 Hospital for Tropical Diseases, Ho Chi Minh City, 700000, Vietnam; 3 School of Cellular and Molecular Medicine, University of Bristol, Bristol, UK; 4 University of Warwick, Coventry, UK; 5 University of Cambridge, Cambridge, UK; 6 Centre for Tropical Medicine and Global Health, Oxford University, Oxford, UK

**Keywords:** dengue, metformin, obesity, clinical trial, therapeutic, Vietnam

## Abstract

**Background:**  Dengue is a disease of major global importance. While most symptomatic infections are mild, a small proportion of patients progress to severe disease with risk of hypovolaemic shock, organ dysfunction and death.  In the absence of effective antiviral or disease modifying drugs, clinical management is solely reliant on supportive measures. Obesity is a growing problem among young people in Vietnam and is increasingly recognised as an important risk factor for severe dengue, likely due to alterations in host immune and inflammatory pathways. Metformin, a widely used anti-hyperglycaemic agent with excellent safety profile, has demonstrated potential as a dengue therapeutic *in vitro* and in a retrospective observational study of adult dengue patients with type 2 diabetes.  This study aims to assess the safety and tolerability of metformin treatment in overweight and obese dengue patients, and investigate its effects on several clinical, immunological and virological markers of disease severity.

**Methods:** This open label trial of 120 obese/overweight dengue patients will be performed in two phases, with a metformin dose escalation if no safety concerns arise in phase one. The primary endpoint is identification of clinical and laboratory adverse events.  Sixty overweight and obese dengue patients aged 10-30 years will be enrolled at the Hospital for Tropical Diseases in Ho Chi Minh City, Vietnam. Participants will complete a 5-day course of metformin therapy and be compared to a non-treated group of 60 age-matched overweight and obese dengue patients.

**Discussion:**  Previously observed antiviral and immunomodulatory effects of metformin make it a promising dengue therapeutic candidate in appropriately selected patients. This study will assess the safety and tolerability of adjunctive metformin in the management of overweight and obese young dengue patients, as well as its effects on markers of viral replication, endothelial dysfunction and host immune responses.

**Trial registration:** ClinicalTrials.gov: NCT04377451 (May 6 ^th^ 2020).

## Background

Dengue is a major threat to global public health, with an estimated annual incidence of 390 million infections worldwide, leading to approximately 96 million symptomatic cases and 22,000 deaths in more than 120 countries ^1^. Despite increasing understanding of dengue pathogenesis, effective anti-viral or disease modifying agents are yet to be identified, and patient management remains reliant on judicious fluid replacement in response to plasma leakage during the critical phase of illness (usually days 4–6) ^2, 3^.

While the vast majority of symptomatic infections give rise to the milder form of disease, dengue fever, those that progress to severe disease carry significant risk of vital organ dysfunction and death. Several patient groups have been identified as being at particular risk of severe dengue, including the elderly, pregnant women, those with comorbid illness, and those with prior exposure to alternative dengue serotypes ^4^. An important and increasingly recognised severe dengue risk factor is obesity, a condition which has had a dramatic increase in prevalence among Vietnamese children and adolescents over the last two decades ^5^. Several observational studies have reported an association between obesity and severe dengue ^6– 9^. A recent systematic review and meta-analysis reported the odds of developing severe dengue to be significantly higher in obese children in comparison to their non-obese counterparts (OR = 1.38; 95%CI 1.10 - 1.73) ^10^.

The underlying mechanism remains unknown, but could possibly be explained by chronic low-grade inflammation that is induced by being overweight or obese ^11, 12^. Adipocytes are known to produce pro-inflammatory cytokines and adipokines, such as adiponectin, resistin and leptin, each of which have a role in immunomodulation. Obesity-induced leptin and resistin are known to be associated with many of the physiological abnormalities seen in dengue, including platelet dysfunction, vascular inflammation, myocardial injury, endothelial dysfunction ^13– 16^, and capillary hyper-permeability ^17– 19^. Additionally, it is possible that more severe infection is permitted by altered CD8 ^+^ T cell immunity and impairment of natural killer (NK) cell activity in obese hosts ^20, 21^.

Adenosine Monophosphate (AMP)-Activated protein kinase (AMPK) is an enzyme involved in maintaining cellular energy homeostasis, in particular lipid metabolism, protein synthesis and glucose metabolism, and has been linked to anti-inflammatory processes ^22^. Obesity has been shown to down-regulate AMPK, either as a result of fatty acid, amino acid and glucose accumulation ^22^, or due to an increase in circulating pro-inflammatory cytokines ^22, 23^. One consequence of AMPK down-regulation is the inhibition of lipid catabolism and subsequent boosting of *de novo* lipid synthesis, creating conditions that favour viral replication and survival within host cells ^24, 25^.

With the majority of symptomatic dengue infections leading to mild, self-limiting illness, development of novel treatment strategies should focus on patient groups at the highest risk of severe disease ^26^. This study aims to assess the effect of metformin, a drug known to attenuate inflammatory processes particularly in the context of obesity, on the disease course of dengue in overweight and obese children and young adults.

### Metformin

Metformin (dimethybiguanide) is an oral anti-hyperglycaemic agent. Its mechanism of action is complex and only partially understood, but AMPK activation is recognised as one of a number of key processes in modulating glucose metabolism ^27^. After sixty years of use as the first-line drug to manage type 2 diabetes mellitus (T2DM), metformin has proven to have an excellent safety and tolerability profile, while also demonstrating potential to have further therapeutic applications through its broad range of pleiotropic effects. These include antioxidant, anti-inflammatory and immunomodulatory properties, as well as aiding endothelial vascular reactivity and preservation of the glycocalyx layer ^28– 30^.

Evidence regarding the effectiveness of metformin in treating obesity is well established. The drug has demonstrated utility in inducing both significant weight loss and a reduction in the inflammatory biomarkers interferon-γ, total plasminogen activator inhibitor-1 (PAI-1) and the adiponectin–leptin ratio in obese adults with or without T2DM ^31– 33^.

Other studies have shown improved clinical outcomes with metformin use in a number of infectious diseases, including hepatitis C (HCV), tuberculosis and dengue ^34– 39^. Notably, the use of metformin as an adjunct in the treatment of patients with HCV and insulin resistance has been shown to improve rates of sustained virological response in comparison to standard anti-viral treatment alone ^40^. Furthermore, two prospective trials of metformin use in patients with HCV and T2DM have demonstrated significant reductions in liver-related complications ^35, 36^.

### Metformin and dengue

Several of the aforementioned effects of metformin treatment have prompted consideration of its use as a potential dengue therapeutic. Firstly, the replication of dengue virus has been shown to be restricted by inhibition of lipid synthesis through AMPK activation ^24, 41^. Furthermore, metformin’s immunomodulatory effect, which facilitates NK cell activation, reduces T cell exhaustion profiles and enhances CD8 ^+^ T cell memory, could help reduce levels of peak dengue viremia and shorten viral clearance times ^39, 41, 42^.

Metformin is rapidly absorbed and eliminated as an oral formulation and steady state is usually achieved within 24 hours. Metformin has been shown to decrease dengue viral genome copies in a dose-dependent manner *in vitro* via activation of AMPK, with 90% reduction in NS1 secretion in infected cells after 24 hours of treatment ^41^. In obese/overweight adolescents, time to peak concentration and absorption, appears to be faster than non-obese children ^43^. In addition, anti-viral effects using a 5 day course of metformin has also been demonstrated in a mouse model of coxsackievirus B3 (CVB3) infection, through rapid action on AMPK activation ^44^.

Prevention of progression to severe dengue by attenuating the inflammatory mediators leptin and resistin is also plausible ^45^. Additionally, metformin could help reverse endothelial dysfunction, a hallmark feature of dengue pathogenesis, by improving endothelial-dependent vasodilation and reducing expression of endothelial vascular cell adhesion molecule (VCAM) and intercellular adhesion molecule 1 (ICAM1) ^24^. Recent observational evidence suggests metformin use in adult dengue patients with T2DM is associated with reduced risk of developing severe dengue (adjusted RR = 0.60, 95%CI 0.37-0.98) ^37^. However, the mechanisms underlying this association, and any relationship they have to obesity, require further investigation.

### Aims of the trial

We hypothesize that metformin therapy given early in the course of disease will attenuate obesity-induced lipid-inflammatory mediators and improve clinical parameters in dengue patients with obesity. In addition, the therapy could reduce viral replication through AMPK activation and immunomodulation in such patients.

This study aims to formally assess metformin as adjunctive therapy for dengue in overweight and obese patients.

## Methods/design

This is protocol version 4.0, dated February 10 ^th^ 2020.

### Design

This is an open-label study investigating metformin therapy in 60 overweight and obese Vietnamese adults (30 patients) and children (30 patients). Age range of 10 to 30 years, who are admitted to the Hospital for Tropical Diseases (HTD) in Ho Chi Minh City (HCMC), Vietnam, with positive non-structural protein 1 (NS1) rapid test and onset of fever <72 hours prior to admission.

We will investigate the effects of five days of metformin treatment on the grounds that the critical phase of disease usually occurs around the fifth day of illness.

**Figure 1.**
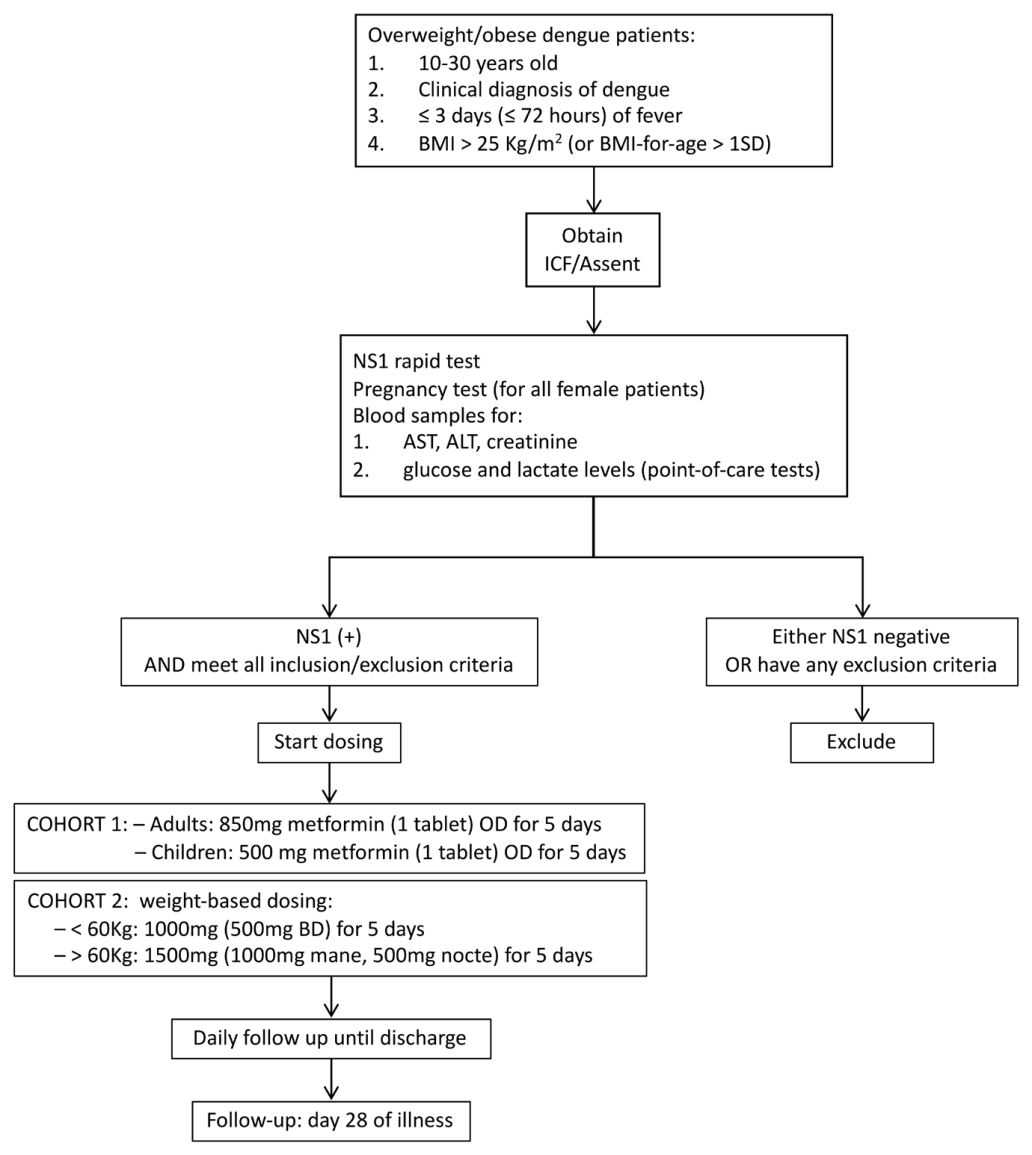
Trial flow chart.

The trial will be conducted in two phases, with a dose escalation between phases ( Figure 1). In the initial phase (cohort 1), five young adults (age ≥16) and five children (age <16) with body mass index (BMI) >25 kg/m ^2^ (BMI-for-age >1 standard deviation – SD) will be provided with a low dose of metformin once daily at 850mg and 500mg, respectively. If the five-day safety and clinical data of cohort 1 show no safety concerns, the study will progress to the second phase (cohort 2). This will include 25 adults and 25 children, who will be given a weight-based dose of metformin; 1000mg (500mg twice daily) for participants with weight <60kg, and 1500mg (1000mg mane, 500mg nocte) for those ≥ 60kg. Of note, the actual number of patients in each cohort will depend on the results of safety reviews. Recruitment is expected to happen over two dengue seasons, and discharged patients will be asked to return for a single outpatient follow-up (FU) visit around day 28 of illness.

The 60 patients receiving treatment will be compared to 60 overweight or obese patients with standard of care (without treatment) recruited contemporaneously from the same wards, with the same enrolment criteria, in an ongoing observational study as the control arm.

### Inclusion and exclusion criteria

Patients will be eligible for study enrolment if they: (i) are aged between 10 and 30 years; (ii) have a clinical diagnosis of dengue (based on World Health Organization (WHO) – Dengue guidelines) ^2^; (iii) are NS1 rapid antigen test positive; (iv) have BMI >25kg/m ^2^ (or BMI-for-age >1 SD); (v) are admitted within 72 hours of fever onset; (vi) agree to come back for FU visit between days 21–28 of illness; and (vii) provide written informed consent.

Exclusion criteria include: (i) pregnancy (confirmed by either clinical examination or urine dipstick for human chorionic gonadotrophin hormone); (ii) localising features suggestive of an alternative diagnosis; (iii) significant diarrhoea and/or vomiting (>3 episodes/24 hours); (iv) history of hypersensitivity to metformin; (v) current use of metformin or any other regular hypoglycaemic agent, including insulin; (vi) current use of any drug with significant metformin interaction ( *Extended data* ^46^); (vii) current treatment for heart failure or recent history of myocardial infarction (in the last year); (viii) presence of severe infection at enrolment, including severe dengue, central nervous system infection or septicaemia; (ix) baseline lactate level >2.0 mmol/L; (x) baseline blood glucose level <3.9 mmol/l (or <70 mg/dL); (xi) baseline liver impairment (alanine transaminase (ALT) and/or aspartate transaminase (AST) >250 U/L); and (xii) chronic renal impairment (glomerular filtration rate (GFR) <30 mL/min). Patients that are deemed unlikely to attend FU at 28 days from fever onset because of long travelling distance to the clinic will also be excluded.

### Primary endpoint

The primary endpoint of this study is an evaluation of the safety and tolerability of metformin therapy in overweight and obese young adults and children with dengue. The number of clinical and laboratory adverse events (AEs) occurring during the five study days ( Table 1), will be compared between the treatment population and the non-treated controls.

**Table 1.** Adverse events.

1- Worsening level of consciousness (fall in GCS ≥ 2 points)
2- Change in blood pressure (PP < 20 mmHg, SBP < 90 mmHg or unmeasurable BP)
3- Vomiting (≥ 3 episodes separated by 15 minutes/ 24 hours)
4- Epigastric abdominal pain
5- Diarrhoea (≥ 5 episodes/24 hours)
6- Mucosal bleeding
7- Rash, urticaria
8- Increased lactate (≥ 3 mmol/L)
9- Episode of hypoglycaemia (glucose < 3.9 mmol/L or < 70 mg/dL)
10- Increased liver enzyme (AST or ALT > 400 U/L)
11- Acute kidney injury (eGFR reduction of >50% or < 30 ml/mn/1,73m ^2^ or plasma creatinine >2 fold increase from baseline)
12- Thrombocytopenia (Platelet < 20.000/uL)
13- Other event, specify: [________________________________________]
**Grade of the event**
Mild: Discomfort noticed but no disruption of normal daily activities
Moderate: Discomfort sufficient to reduce or affect daily activity
Severe: Inability to perform daily activity

### Secondary endpoints

The secondary endpoints are: fever clearance time (the time from enrolment to sustained body temperature <37.5°C for 48 hours); platelet nadir and highest AST/ALT between days 3 and 8 of illness; percentage increase in haematocrit (HCT) from baseline (using HCT obtained at the 28 day FU visit to reflect baseline); plasma viraemia (area under the log _10_-transformed viraemia curve from study days 3 to 6); time from enrolment to first undetectable viraemia and first negative NS1 measurement; trend over time (5 days) in VCAM1, CRP, leptin, adiponectin, low density lipoprotein (LDL), and AMPK phosphorylation; and magnitude of activated/proliferating T and NK cells as well as frequency and phenotype of dengue-specific CD4+ and CD8+ T cells, quantified by flow cytometry at enrolment and hospital discharge.

### Screening, enrolment and retention

Patients aged between 10 and 30 years that are admitted to one of the three study wards of the HTD, HCMC, Vietnam, with clinical suspicion of dengue and less than 72 hours of fever are invited to participate in the trial. After giving consent, patients are screened for their eligibility to commence treatment with the trial drug by the study physicians. Blood samples are collected to test for NS1, and AST, ALT and creatinine levels and in female patients, a urine sample is collected for a pregnancy test. Glucose and lactate levels are measured using point-of-care (POC) tests.

All patients will be asked to come back for a final FU visit at 28 days after the onset of fever. Patients will be contacted 3–5 days prior to the FU visit to arrange an appropriate appointment date and time. Any case who misses the FU visit will be contacted by phone either to reschedule the visit or to collect information if patients could not arrange their time to come back. If the patients stay in the hospital for longer than 5 days, due to AEs or any other reason, the treatment for these cases will be at discretion of the attending physicians. No further blood samples or study procedures will be performed from this point onward. However, the final outcome at discharge will be collected in the case report form (CRF; *Extended data* ^46^).

At discharge, the participants will be offered a referral to nutrition specialists for a consultation of overweight/obesity management. Patients will also be provided with leaflets on healthy eating and lifestyle behaviours.

While not an RCT, this trial is designed to enroll patients contemporaneously with an observational obesity trial, where patients received standard care, to minimize systematic differences between the intervention (metformin) and control group, i.e. patients have the same inclusion /exclusion criteria, enrolled on same wards, with the same standard of care during the same dengue season (to avoid the issue of viral serotype shifts). 

To minimize selection bias, we have set up a system on the dengue wards, to ensure eligible patients are recruited into both studies in a systematic way. This involves only one study enrolling for a 2-week time block. During that time, if a patient fails screening assessment or declines enrollment into one of the trials, they are not eligible for the other.

In addition, we will ensure the predefined inclusion/exclusion criteria for all patients enrolled are strictly adhered to reduce investigator subjective assessment and will carefully document screen failures and all exclusions and withdrawals in the trial. And lastly, we plan to have an intention-to-treat analysis where independent study investigators will perform a blinded outcome assessment for all patients.

### Safety reviews

The trial will be conducted in two phases, with a dose escalation if no safety concerns arise during the low dose stage. Metformin dosing will begin at 500mg (children) and 850mg (adults) once daily for 5 days in cohort 1. Daily patient safety reviews will be performed by study doctors using clinical and laboratory data to decide whether patients are safe to continue taking the study drug.

The study drug will be stopped if participants: (1) request withdrawal from the study; (2) develop any AEs, including severe renal impairment (GFR below 30mL/min/1.73m ^2^), elevated lactate to ≥ 3 mmol/L, severe liver involvement (AST/ALT > 400U/L), severe diarrhoea (≥ 5 episodes of loose stool/day), persistent vomiting (≥ 3 episodes/hour or ≥ 4 / 6hours) and hypoglycaemia (blood glucose < 3.9 mmol/l or < 70 mg/dL); (3) develop severe dengue (defined as admission to intensive care unit); or (4) are intolerant of metformin.

All AEs occurring during the trial/or until the FU visit at around day 28 of illness, will be recorded in the CRF, and whether or not attributed to trial medication. Specific AEs are shown in Table 1. Laboratory events will be graded according to Common Terminology Criteria for Adverse Events (CTCAE) definitions. Clinical events will be assessed as mild, moderate, or severe. If the event is serious and not only related to the progression to severe dengue, or is fatal, then a serious AE form must be completed and the OUCRU CTU notified within 24 hours.

In cases of discontinuation due to AEs, participants will be followed up until the events have resolved or stabilised. A Data Monitoring Committee (DMC) review will take place after day 5 data is fully available for the first ten patients enrolled in cohort 1. Following satisfactory safety review of the tenth patient, metformin doses for all remaining patients (cohort 2) will be weight-based; 5 days of 1000mg (500mg twice daily) for those weighing <60kg and 5 days of 1500mg (1000mg mane, 500mg nocte) for those weighing ≥60kg. All AEs and serious adverse events (SAEs) will be recorded and reported to the Ethics Committees (ECs) for review. Further DMC meetings will take place 6 monthly to review enrolment, AEs, treatment received and FU information, and any other requested data.

### Treatment and drug dispensation

Metformin will be kept in a dry location below 30°C, in a secure area. A study pharmacist will make up and label individual-participant treatment packs with a trial number. Each pack contains sufficient study drug for 5 days of treatment and will be distributed to study wards as required. All medication storage and administration will be regulated through the central pharmacy to ensure good quality and control of medication handling.

Patients will be recruited into either cohort 1 (low dose) or cohort 2 (high dose). Study drugs will be administered as directly-observed-therapy. Patients will take the first dose of study drug with a light snack as soon as possible after enrolment. The remaining doses will be taken after meals. In case of vomiting within 30 minutes of taking the treatment, one replacement dose will be given. However, if patients continue vomiting after taking the replacement dose, no extra drug will be given thereafter.

### Data collection

***Clinical evaluation.*** Patients will be reviewed daily until discharge. All clinical symptoms and signs will be recorded in the CRF. An ultrasound scan will be performed on day 5–6 of illness to detect evidence of fluid accumulation. General management decisions will be at the discretion of the attending ward doctors. Details of all AEs and SAEs will be recorded on specific forms, together with an assessment as to whether the events are likely to have been related to any treatment received. All SAEs will be reported promptly to the DMC and ECs according to policy.

***Laboratory evaluation.*** Full blood count (FBC) and POC glucose and lactate tests will be undertaken daily until discharge. An additional POC glucose test will be performed before their evening meal.

In addition, levels of serum albumin, transaminases (ALT/AST), creatinine, total cholesterol, HDL-cholesterol, LDL-cholesterol, triglycerides, and C-reactive protein (CRP) will be measured on alternate days. All tests will be done more frequently when clinically indicated.

Research samples collected daily until discharge will be used for dengue diagnostics (NS1 antigen detection and PCR assays). Residual blood from FBC samples will be collected for daily plasma viraemia levels. Serology tests (Capture IgM/IgG ELISAs) will be performed on enrolment (acute) and discharge/FU (convalescent) samples. Other laboratory investigations will include endothelial, inflammatory and lipid biomarkers (VCAM1, syndecan-1, leptin, adiponectin), using commercially available ELISAs and Luminex kits, immunological tests (T cell phenotype and functional analysis), and immunometabolism (AMPK phosphorylation). RNA will be extracted from a blood sample collected on study day 2, and will be sequenced to investigate gene expression in the metformin treatment group in comparison to the non-treatment group.

At day 28 of illness, final routine (FBC, biochemistry) and research blood samples will be taken to check whether patients have fully recovered from the acute dengue episode.

### Statistical considerations

***Sample size.*** This is an exploratory study focusing primarily on safety, and there are no preliminary data regarding the effects of metformin in dengue on which to base a sample size calculation. A target sample size of 60 patients was chosen based on clinical judgement and feasibility considerations. Using the trend in VCAM over time, which is one of the secondary endpoints, by assuming a slope of change in concentration over time at 0.34 (log _2_VCAM concentration per day, data based on biomarkers of endothelial function in dengue), we conducted a power calculation using a t-test to compare effects on endothelial function between two groups. It is suggested this study will have a power of 80% to detect a difference of at least 0.023 (log _2_VCAM concentration per day) in the slope of change.

***Statistical analysis.*** The primary analysis population will include all patients from cohort 1 and cohort 2, plus 60 controls as mentioned previously. Baseline characteristics and outcomes will be summarised for each treatment group. The cohort of patients enrolled in this study will form the active treatment group, while the control group will consist of overweight and obese dengue patients that participated in a matched cohort observational study, none of whom received metformin therapy. Continuous variables will be described using medians and quartiles, while categorical variables will be described in terms of frequencies and percentages.

The primary endpoint (number of AEs), will be compared between the treatment groups using negative binomial regression model. The clinical, biomarker, immunological will be compared between the treatment arms using linear regression or random effects model for continuous endpoints, and Cox regression for time-to-event endpoints (fever clearance time, time to first undetectable viremia measurement, time to first negative NS1 measurement). All comparisons will be adjusted for age, sex, and day of illness, as patients will not be randomized. For percentage increase in HCT and area under the curve of viraemia, comparisons will be also adjusted for the pre-dose value of the respective marker.

## Ethical considerations

### Ethical approval

The protocol, informed consent forms, assent forms and patient information sheets have been reviewed and approved by the ECs of the Hospital for Tropical Diseases in Ho Chi Minh City (CS/BND/19/34), the Vietnamese Ministry of Health (24/CN-HÐÐÐ) and the Oxford Tropical Research Ethics Committee (OxTREC reference: 36-19). The Investigator will submit and, where necessary, obtain approval from the above committees for all amendments to the originally approved documents.

### Informed consent and information sheet

All patients entering the study must give written informed consent. The study physician will describe the purpose of the study, the study procedures, possible risks/benefits, the rights and responsibilities of participants, and alternatives to enrolment. The patient or parent/guardian will be invited to ask questions which will be answered by study staff, and they will be provided with appropriate numbers to contact if they have any questions subsequently. If the patient or parent/guardian agrees to participate, they will be asked to sign informed consent/assent forms (ICFs; *Extended data* ^46^). A copy of the form, with signature, will be given to them to keep. In addition to the procedures above, illiterate signatories will have the ICF read to them in the presence of a witness who will sign to confirm this.

Patients, who are between 10 and 18 years of age, will be asked to sign the assent form in which, the study purpose and procedures will be explained to them in child friendly terms. Their parent/guardian will be asked to sign the consent form as well to give permission for their child to participate in the study.

### Confidentiality

Patients who enter the trial will be given a unique identification number. This number will be used on both laboratory specimens and CRFs. The study wards and the research unit have the facilities to store study information securely. Only study staff will have access to the password-protected computer where entered data is stored. After conclusion of the study, data will be stored in a safe place. Any scientific publications or reports will not identify any patient by name or initials. When the research team reviews the clinical notes, they are also bound by professional confidentiality.

### Data monitoring and trial steering committees

A Trial Steering Committee (TSC) will be formed to provide overall supervision of the conduct of the trial and provide advice through its independent Chair. The ultimate decision for the continuation of the trial lies with the TSC. In particular, the TSC will concentrate on progress of the trial, adherence to the protocol, patient safety, and the consideration of new information.

An independent DMC will be set up consisting of qualified volunteers with the necessary knowledge of clinical trials and statistics. The DMC will review the protocol and agree to a data review schedule and reporting requirements before the study commences. All data reviewed by the DMC will be in the strictest confidence. A DMC charter will outline its responsibilities and operations ( *Extended data* ^46^).

Monitoring will then be carried out approximately annually by OUCRU CTU staff. The frequency, type and intensity of routine monitoring and the requirements for triggered monitoring will be detailed in the monitoring plan, which will also detail the procedures for review and sign-off. The monitoring will adhere to the principles of ICH GCP and the monitoring plan.

### Data collection and management

All study data will be recorded on standard CRFs and entered on the CliRes database. The participants will be identified by a unique study specific number and/or code in any database. The name and any other identifying detail will not be included in any stored electronic CRFs. Direct access will be granted to authorized representatives from the University of Oxford and any host institution for monitoring and/or audit of the study to ensure compliance with regulations. Original paper documents will be stored at the OUCRU-CTU for 15 years after which paper files will be scanned and archived electronically. Electronic data will be stored indefinitely on a secure OUCRU-VN server for a minimum period of 10 years and paper copies will be destroyed securely.

## Spirit checklist

A SPIRIT checklist for this trial protocol is attached ( *Reporting guidelines* ^46^).

## Data sharing and dissemination

In line with research transparency and greater access to data from trials, OUCRU’s clinical trials are registered at ClinicalTrials.gov and a data sharing policy is in place. Data exchange complies with Information Governance and Data Security policies in all of the relevant countries.

Participants will not be individually informed of results. Data from this study will be published in peer-reviewed journals. Authorship and reporting of this work will follow international guidelines and all authors will have made a noteworthy contribution to the work.

## Trial status

We expect that this trial will start recruiting patients in June 2020.

## Discussion

Despite increasing investment in strategies to prevent infection, dengue remains a major global pathogen, with extremely high associated health, social and economic disease burden ^47^. Severe dengue can be rapidly life threatening as a result of plasma leakage, hypovolaemic shock and subsequent end organ damage, and is known to disproportionately affect overweight and obese individuals. Although optimal supportive care is largely successful in permitting recovery from most dengue infections, effective therapeutic agents are desperately needed to enable treatment of patients with the greatest disease severity. Metformin has previously demonstrated therapeutic potential due to observed anti-viral and immunomodulatory effects, and an association with reduced risk of severe dengue in adults with T2DM. Additionally, its low cost and excellent safety profile in the treatment of T2DM make metformin a prime candidate for use in appropriately selected patients on a mass scale. This study will assess the safety and tolerability of metformin in the context of dengue infection, and whether metformin treatment early in the course of disease is clinically beneficial for overweight and obese children and young adults. It will also provide a key insight into the effects of metformin on markers of viral replication, endothelial dysfunction and the host immune response in this patient group. If the safety of this drug is established and this pilot study demonstrates improvement in any of the secondary endpoints, a larger randomized-controlled trial of metformin in dengue would be warranted

## Data availability

### Underlying data

No underlying data are associated with this article.

### Extended data

Oxford University Research Archive: Oxford University Clinical Research Unit Metformin trial supporting documents, https://doi.org/10.5287/bodleian:5Rk2p9EVK ^46^.

- Consent form
- Participant information sheet
- DMC charter
- Case Report Form
- Drug interactions

### Reporting guidelines

Oxford University Research Archive: SPIRIT checklist for ‘Metformin as adjunctive therapy for dengue in overweight and obese patients: a protocol for an open-label clinical trial (MeDO)’, https://doi.org/10.5287/bodleian:5Rk2p9EVK ^46^.

Data are available under the terms of the Creative Commons Attribution 4.0 International license (CC-BY 4.0).
