# Supplementary material for: Metformin as adjunctive therapy for dengue in overweight and obese patients: a protocol for an open-label clinical trial (MeDO)
Source: Wellcome Open Res. 2021 Feb 8;5:160. Originally published 2020 Jul 6. [Version 2] doi: 10.12688/wellcomeopenres.16053.2 (PMC7539082; doi:10.12688/wellcomeopenres.16053.2)
Supplement: Peer review history

# Peer review: Reviewer response for version 2

**DOI:** 10.21956/wellcomeopenres.18074.r42516

**Published:** 2021-02-24

**Authors:** Rosa María Del Ángel

**Affiliations:** 1 Department of Infectomics and Molecular Pathogenesis, Center for Research and Advanced Studies (CINVESTAV-IPN), Mexico City, Mexico

This revised version of the manuscript is now suitable for indexing.

Is the study design appropriate for the research question?

Yes

Is the rationale for, and objectives of, the study clearly described?

Yes

Are sufficient details of the methods provided to allow replication by others?

Partly

Are the datasets clearly presented in a useable and accessible format?

Yes

Reviewer Expertise:

NA

I confirm that I have read this submission and believe that I have an appropriate level of expertise to confirm that it is of an acceptable scientific standard.

# Peer review: Reviewer response for version 1

**DOI:** 10.21956/wellcomeopenres.17609.r40457

**Published:** 2020-10-08

**Authors:** Rosa María Del Ángel, Juan Fidel Osuna-Ramos

**Affiliations:** 1 Department of Infectomics and Molecular Pathogenesis, Center for Research and Advanced Studies (CINVESTAV-IPN), Mexico City, Mexico; 2 Centro de Investigacion y de Estudios Avanzados (Cinvestav), Mexico City, Mexico

This manuscript reports the rationale and design of a clinical trial to test the safety of a 5-day course of metformin as treatment to reduce the likelihood of severe disease in obese dengue patients. The motivation of this study is the emerging epidemiological association between obesity and increased risk of severe dengue. The authors reasoned that activation of AMPK by metformin would counteract the reduced AMPK activity in obese patients; lowered AMPK is associated with altered lipid metabolism that increase dengue virus (DENV) replication, *in vitro*. Thus, the authors postulate that activation of AMPK by metformin would result in an increase in lipid catabolism inhibiting DENV replication and viremia, which has been found in several studies to be correlated with disease outcome. They also postulate that metformin would also exert effects on the immune response, including NK cell activation and reduction in T-cell exhaustion, that would collectively contribute to limiting DENV infection and replication. To test the hypothesis, the authors' first aims to show that high-dose metformin can be given safely to patients acutely ill with dengue; standard doses of metformin would be administered to ensure safety before dose escalation. The primary endpoint of the study is to observe the safety and tolerability of metformin in overweight and obese young adults and children with dengue.

 This is a timely study as the prevalence of both obesity and dengue are increasing globally. There are several areas of consideration.

1. It has been described that viremia in most patients after illness onset decline rapidly. Thus impact of metformin on AMPK activation must be rapid. How much time is necessary to induce the activation of AMPK by metformin? Please include a discussion about this aspect.
2. If obesity is a risk factor for dengue infection. Why in this clinical trial enrollment of non-obese persons is not considered?
3. Why did the authors select 28 days?
4. Why is placebo not going to be included?
5. DENV infection will be determined by the detection of NS1. How frequent is the infection with other flavivirus in the region? Is the detection of NS1 specific to confirm DENV infection? The authors could use RT-PCR instead of NS1 to confirm DENV infection.
6. How are the authors going to measure VCAM1, CRP, leptin, adiponectin, low density lipoprotein (LDL), and AMPK phosphorylation?

Minor comment:

1. GRF on page 5 should be replaced with GFR.

Is the study design appropriate for the research question?

Yes

Is the rationale for, and objectives of, the study clearly described?

Yes

Are sufficient details of the methods provided to allow replication by others?

Partly

Are the datasets clearly presented in a useable and accessible format?

Yes

Reviewer Expertise:

Antiviral in flavivirus infection

We confirm that we have read this submission and believe that we have an appropriate level of expertise to confirm that it is of an acceptable scientific standard, however we have significant reservations, as outlined above.

# Response

**Published:** 2020-11-02

**Authors:** Nguyet Nguyen

**Affiliations:** Oxford University Clinical Research Unit, Vietnam

Comment #1: Thank you for this suggestion, we also acknowledge that there is a narrow therapeutic window, in all dengue therapeutic trials. We are therefore aiming to give the drug within the first 48 hours of fever (latest 72 hours). Metformin is rapidly absorbed and eliminated as an oral formulation and steady state is usually achieved within 24 hours. In obese/overweight adolescents, time to peak concentration and absorption, appears to be faster than non-obese children {van Rongen A. et al. 2018. *Paediatr Drugs*}.   An in-vitro study showed metformin increased AMPK activity after 24 hours and reduced of dengue viral replication by one logarithm and up to 90% of NS1 secretion {Soto-Acosta R. et al. 2017. *PLoS Pathog*}. In addition, anti-viral effects using a 5 day course of metformin has also been demonstrated in a mouse model of coxsackievirus B3 (CVB3) infection, through rapid action on AMPK activation {Xie W. et al. 2015. *J Mol Cell Cardiol*}.

 The effect of metformin on AMPK and reduction in viremia is just one mechanism why we hypothesize this drug will be beneficial in dengue patients, its other effects, including anti-inflammatory, immunomodulatory and endothelial stabilizing properties  are also likely to be important mechanisms in attenuating the disease outcome.

 A paragraph has been added to "Metformin and Dengue" section.

 Comment #2: We chose to focus on obese patients initially, as these patients have a higher risk of disease progression and therefore warrant an intervention. In addition, we hypothesize many of metformin actions, including anti-inflammatory, immunomodulatory, endothelial stabilizer and AMPK activation are already chronically impaired at baseline in obese individuals, thus maximizing the chance of demonstrating efficacy in this population.

 Comment #3: The follow-up time point of 28 days is to allow full recovery from the acute illness, and is in line with other clinical trials.

 Comment #4: This is a pilot study investigating the safety and tolerability of dengue obese patients to metformin. If the safety of this drug is established, we plan to move to a phase 3 RCT, with larger sample size to allow investigation of clinical endpoints.

 Comment #5: Other flaviviruses do circulate in Vietnam including Zika and Japanese encephalitis but these are rare and do not cause the same clinical syndrome as dengue {Quyen NTH et al. 2017. *Am J Trop Med Hyg*}. The sensitivity of NS1 tests for dengue varies by immune status, illness day and serotype ranging from 62-89% , but are highly specific (around 100%) {Tricou V. et al. 2011. *PLoS Negl Trop Dis*; **Hermann LL et al. 2014. *PLoS Negl Trop Dis*}

 As RT-PCR is costly and time consuming, it is not ideal as a screening test- we are however confirming diagnosis at a later stage with RT-PCR and paired serology.

 Comment #6: VCAM1, CRP, leptin, adiponectin, low density lipoprotein (LDL), and AMPK phosphorylation will be measured using a luminex platform and commercially available ELISAs.

 This has been added to the laboratory evaluation section.

# Peer review: Reviewer response for version 1

**DOI:** 10.21956/wellcomeopenres.17609.r40522

**Published:** 2020-10-06

**Authors:** Quirijin de Mast

**Affiliations:** 1 Department of Internal Medicine, Radboudumc Center for Infectious Diseases, Radboud University Medical Centre, Nijmegen, The Netherlands

This manuscript describes the rationale and design of a trial to investigate the safety and tolerability of metformin as adjunctive therapie in obese children and adults with dengue. Secondary endpoints include dengue-specific (e.g. fever clearance time, viremia, blood markers of severity) and immune parameters 

 The manuscript is well written. The rationale and design are clear. 

 Areas for consideration are:

- the trial is not placebo controlled. The authors may consider explaining in more detail why they opted for an open label trial without randomisation
- dengue is an acute infection and any intervention should preferably have an immediate effect. The onset of action of metformin on blood sugars usually takes some days with a maximum effect of up to 2 weeks. The authors may consider arguing in more detail that the onset of the expected pharmacological effect of metformin is sufficiently rapid to have an effect on the course of dengue

Is the study design appropriate for the research question?

Yes

Is the rationale for, and objectives of, the study clearly described?

Yes

Are sufficient details of the methods provided to allow replication by others?

Yes

Are the datasets clearly presented in a useable and accessible format?

Yes

Reviewer Expertise:

clinical infectious diseases; pathophysiology studies

I confirm that I have read this submission and believe that I have an appropriate level of expertise to confirm that it is of an acceptable scientific standard.

# Response

**Published:** 2020-11-02

**Authors:** Nguyet Nguyen

**Affiliations:** Oxford University Clinical Research Unit, Vietnam

Comment #1: This is a pilot study investigating the safety and tolerability of metformin in dengue patients. As metformin has not been used in acute dengue infections before, this trial was designed to establish safety first, as well as generate some clinical and viral efficacy data. A larger phase 3 RCT will be conducted if this trial shows a positive result.

 Comment #2: Thank you for this suggestion, we also acknowledge that there is a narrow therapeutic window, in all dengue therapeutic trials. We are therefore aiming to give the drug within the first 48 hours of fever (latest 72 hours). Metformin is rapidly absorbed and eliminated as an oral formulation and steady state is usually achieved within 24 hours. In obese/overweight adolescents, time to peak concentration and absorption, appears to be faster than non-obese children {van Rongen A. et al. 2018. *Paediatr Drugs*}.   An in-vitro study showed metformin increased AMPK activity after 24 hours and reduced of dengue viral replication by one logarithm and up to 90% of NS1 secretion {Soto-Acosta R. et al. 2017. *PLoS Pathog*}. In addition, anti-viral effects using a 5 day course of metformin has also been demonstrated in a mouse model of coxsackievirus B3 (CVB3) infection, through rapid action on AMPK activation {Xie W. et al. 2015. *J Mol Cell Cardiol*}.

 The effect of metformin on AMPK and reduction in viremia is just one mechanism why we hypothesize this drug will be beneficial in dengue patients, its other effects, including anti-inflammatory, immunomodulatory and endothelial stabilizing properties  are also likely to be important mechanisms in attenuating the disease outcome.

 A paragraph has been added to "Metformin and Dengue" section.

# Peer review: Reviewer response for version 1

**DOI:** 10.21956/wellcomeopenres.17609.r39444

**Published:** 2020-07-28

**Authors:** Eng Eong Ooi

**Affiliations:** 1 Emerging Infectious Diseases Program, Duke NUS Graduate Medical School, Singapore, Singapore

This manuscript reports the rationale and design of a clinical trial to test the safety of a 5-day course of metformin as treatment to reduce the likelihood of severe disease in obese dengue patients. The motivation of this study is the emerging epidemiological association between obesity and increased risk of severe dengue. The authors reasoned that activation of AMPK by metformin would counteract the reduced AMPK activity in obese patients; lowered AMPK is associated with altered lipid metabolism that favoured dengue virus (DENV) replication, *in vitro*. They thus postulate that activation of AMPK by metformin would result in increased lipid catabolism that would inhibit DENV and thus lower viremia, which has been found in several studies to be correlated with disease outcome. They also postulate that metformin would also exert effects on the immune response, including NK cell activation and reduction in T cell exhaustion, that would collectively contribute to limiting DENV infection and replication. To test their hypothesis, the authors first aims to show that high-dose metformin can be given safely to patients acutely ill with dengue; standard doses of metformin would be administered to ensure safety before dose escalation. The primary endpoint of the study is to observe the safety and tolerability of metformin in overweight and obese young adults and children with dengue.

 This is a timely study as the prevalence of both obesity and dengue are increasing globally. There are several areas of consideration. 

1. The overview of metformin and its potential impact on dengue pathogenesis, through its activity on the AMPK pathway, has not considered how fast metformin would exert this pharmacologic effect. Viremia levels in most patients after illness onset would be declining rapidly. The impact of metformin on AMPK activation must thus be rapid. Likewise, the effect AMPK-induced lipid metabolism has on DENV replication must also be rapidly induced, if metformin is to have a chance to be repurposed as an anti-dengue drug. Perhaps the authors would consider adding a discussion on this challenge to the manuscript?
2. Phase 1 of the trial will observe the safety and clinical data in 10 dengue patients given a 5-day course of standard doses of metformin, over a 5-day period. Should the safety and clinical data not be observed over a longer period, at least until the drug has washed out before a conclusion of no safety concern is made?
3. This is not a placebo-controlled trial. Neither are the patients randomised into receiving metformin or standard of care. Instead, the authors planned to compare treated patients with contemporaneously enrolled patients. How would the authors address the issue of bias in their patient selection?
4. On a related note, how would the authors address the issue of observer bias without blinding?

Minor comment:

1. GRF on page 5 should be replaced with GFR.

Is the study design appropriate for the research question?

Yes

Is the rationale for, and objectives of, the study clearly described?

Yes

Are sufficient details of the methods provided to allow replication by others?

Yes

Are the datasets clearly presented in a useable and accessible format?

Not applicable

Reviewer Expertise:

Dengue virus-host interactions, pathogenesis and immunity.

I confirm that I have read this submission and believe that I have an appropriate level of expertise to confirm that it is of an acceptable scientific standard.

# Response

**Published:** 2020-11-02

**Authors:** Nguyet Nguyen

**Affiliations:** Oxford University Clinical Research Unit, Vietnam

Comment #1: Thank you for this suggestion, we also acknowledge that there is a narrow therapeutic window, in all dengue therapeutic trials. We are therefore aiming to give the drug within the first 48 hours of fever (latest 72 hours). Metformin is rapidly absorbed and eliminated as an oral formulation and steady state is usually achieved within 24 hours. In obese/overweight adolescents, time to peak concentration and absorption, appears to be faster than non-obese children {van Rongen A. et al. 2018. *Paediatr Drugs*}.   An in-vitro study showed metformin increased AMPK activity after 24 hours and reduced of dengue viral replication by one logarithm and up to 90% of NS1 secretion {Soto-Acosta R. et al. 2017. *PLoS Pathog*}. In addition, anti-viral effects using a 5 day course of metformin has also been demonstrated in a mouse model of coxsackievirus B3 (CVB3) infection, through rapid action on AMPK activation {Xie W. et al. 2015. *J Mol Cell Cardiol*}.

 The effect of metformin on AMPK and reduction in viremia is just one mechanism why we hypothesize this drug will be beneficial in dengue patients, its other effects, including anti-inflammatory, immunomodulatory and endothelial stabilizing properties  are also likely to be important mechanisms in attenuating the disease outcome.

 A paragraph has been added to "Metformin and Dengue" section.

 Comment #2: Thank you - all patients will be followed up at day 28 and will have safety data recorded at that follow-up time-point. For cohort 1 the DSMB have agreed to review day-5 data for the first safety review and all 28-day data for further reviews.

 We do not anticipate any issues of drug accumulation, as in patients with normal renal function, the half life is short and the drug rapidly eliminated (a dose of 1000mg has a Tmax of 1.5 hours and a half-life of 2.3hrs {Chung H. et al. 2018. *PLoS One*}). The drug will be stopped in any patient developing acute renal failure.

 Comment #3 and #4: While not an RCT, this trial was designed to enroll patients contemporaneously with an observational obesity trial, where patients received standard care, to minimize systematic differences between the intervention (metformin) and control group. I.e. patients have the same inclusion /exclusion criteria, enrolled on same wards, with the same standard of care during the same dengue season (to avoid the issue of viral serotype shifts).

 To minimize selection bias, we have set up a system on the dengue wards, to ensure eligible patients are recruited into both studies in a systematic way. This involves only one study enrolling for a 2-week time block. During that time, if a patient fails screening assessment or declines enrollment into one of the trials, they are not eligible for the other.

 In addition, we will ensure the predefined inclusion/exclusion criteria for all patients enrolled are strictly adhered to reduce investigator subjective assessment and will carefully document screen failures and all exclusions and withdrawals in the trial. And lastly, we plan to have an intention-to-treat analysis where independent study investigators will perform a blinded outcome assessment for all patients.

 We have added this to the ‘Screening, enrollment and retention paragraph.’

 Comment #5: Thank you. The typo error has already been corrected
